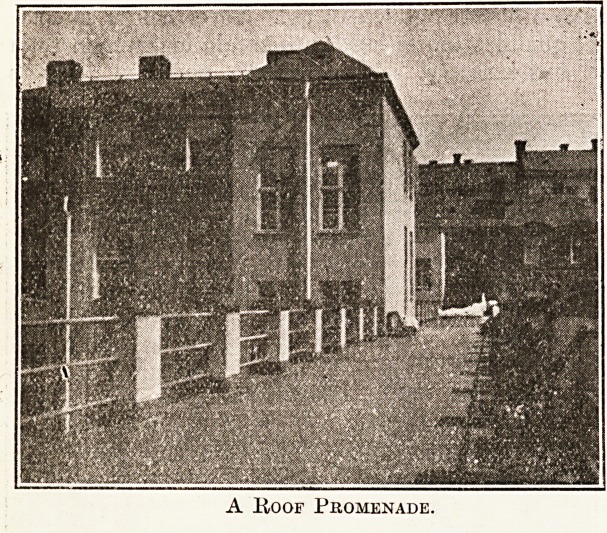# A Visit to Stockholm Hospitals

**Published:** 1915-01-30

**Authors:** 


### I.—A VISIT TO STOCKHOLM HOSPITALS.

### By a Travelling R.M.O.

SWEDEN cannot yet be described as a "tourist-infested" country; even the Germans of the Baltic littoral do not bulk largely among the visitors that one ordinarily meets there, although in peace time excellent services of steamers are run from Lübeck, Sassnitz, etc. Nevertheless inquiries confirm the impression that every recent year has seen both a greater number of visitors and a wider range of nationalities. To Englishmen the direct sea route is undoubtedly a deterrent, and the land route seems too much involved. What the Swedish hotel-keepers should agitate for is a line of much larger and swifter vessels between Hull or Harwich and Gothenburg.

The English traveller in Sweden will be agreeably surprised at the frequency with which he is able to make use of his own language. The reason for this is to be found partly in the fact that large numbers of Swedes have visited and lived in the United States of America, and these are scattered fairly liberally all over the country. In the large centres English or German is almost equally serviceable, but German is not quite so common as might be expected.

### SWEDISH BATHING.

The natural beauties of the country may not here be dilated upon; suffice it to mention that Sweden is the land of lakes and islands—a paradise for sailing boats and motor launches. Around Stockholm, where the sea is separated from Lake Mälar by a tiny barrier and lock, one can hardly see anything like a lake, so numerous are the islands. Indeed the approach to the capital from the Baltic through hundreds of islands is certainly one of the most picturesque trips in Europe. A voyage across Sweden may be made entirely by water, through the large lakes and the Gotha Canal to Gothenburg. On the west coast there are again numerous islands, but these form a direct contrast to those on the Stockholm side, which are wooded, while those around Gothenburg are rugged rocks.

In summer thousands of persons live in a very simple fashion on the islands, where boating and bathing are their chief occupations. Motor boats take the business men to town every day, and everybody who can passes the short but hot summer on some little island or other, especially around Stockholm. Last summer was hotter than usual in most parts of Sweden, and the rocks around the capital were, without exaggeration, swarming with human beings all day long. Complete nudity was the rule, both for men and women, and the latter in small groups were not separated from the former by anything more substantial than a few yards of air. Sun bathing was carried out very thoroughly, both on the rocks and in sailing boats could be seen men whose skins were a darker red than anything that could be produced in this country, no matter how hot the summer.

In the medical world, as one would expect, both practice and teaching have a good deal in common with the German model. In self-interest medical papers are often written in German, and the visitor will find that the foremost medical men all have a good knowledge of German, but a very large number can also display a useful amount of English. The medical curriculum is long and strenuous, and consequently expensive. The status of the medical practitioner is better than at home, because the possession of some private means is almost a necessity in view of the comparatively small fees which rule in general practice. Not a very high opinion is entertained of English medical education and practice, but this need not be taken seriously, for it represents merely second-hand impressions. Those medical men who have studied in England have a higher opinion of us. Nevertheless a medical qualification is harder to obtain in Sweden than in our country. Many of the northern districts are very badly off for doctors, and the medical profession as a whole is not at present overcrowded. Foreigners who desire to practise in Sweden must pass the full curriculum.

THE CORRIDOR.

### THE STOCKHOLM HOSPITALS.

Generally speaking the hospitals of Stockholm are more like ours than those of large German towns—that is to say, the pavilion system is not in evidence and the areas covered by the institutions are modest in extent, though walks and open spaces for the patients are as a rule plentiful enough. The largest hospital in Stockholm is that known as the Sabbatsberg, situated on high ground to the north-west of the town. Though not exactly modern in construction, the hospital is thoroughly well organised and equipped internally. Fine, well-lit corridors conduct the visitor from one portion to another, and there are plenty of lifts. The main hospital buildings are large and lofty blocks, containing wards on their several floors, with nurses' rooms on the topmost storey of some, and operating theatres surmounting other blocks. The wards are grouped in divisions, numbered and lettered, and present quite a familiar appearance to the English visitor. In point of fact one might easily imagine that the wards and the nurses were part of a London hospital. In charge of one of the wards was a sister who had spent over a year in a London hospital, and she admitted that the methods and training in Sweden were not very different from those at home.

### The Independent Sister.

One point, however, was abundantly clear: the position of the sister in charge of a ward is of great independence and responsibility. The sister is in complete control of her unit, and responsible only to the corresponding physician or surgeon. The matron holds an administrative post, but except for a general supervision of the probationers' welfare outside the wards she does not concern herself

A Roof Promenade.

with the work in the wards. To show how completely the sister is in touch with the work of her unit, it must be noted that not only does her sitting-room immediately adjoin the ward, but the same chamber is also her ordinary bedroom! It was all, of course, very comfortable and daintily furnished, but it may well be asked if our sisters would care to be so perpetually on top of their work. At night time it is the recognised thing that the sister shall be called to any serious case, or to do any important piece of nursing. Off-duty time is arranged according to the press of work, and her own freedom is a personal matter; several days may elapse without her leaving the hospital. When the sister is "off," the control of the ward apparently passes to the sister of a neighbouring ward, and not to an equivalent of our staff nurse; the ward would naturally be "light" at such a time. Outside the ward are the usual store-rooms, offices, and small kitchen; in addition there is a private bath-room for the sister, and where an operating theatre exists as on the top floor, there will be a bath- and dressing-room for the surgeon.

### Ward Equipment.

The ward equipment calls for no special mention except as regards the peculiar construction of some of the beds. Those in question had, instead of a spring frame of the usual wire pattern, numerous vertical springs resting on laths, just like those in a sofa or arm-chair; on top of these bare coils the mattress was placed. The dusting of these beds must be somewhat laborious.

A visitor to Sweden will not fail to be impressed by the striking ubiquity of the telephone; the reputation in this respect possessed by Sweden is well known, and Stockholm itself holds the record for the number of telephones per head of the population. The cheapness of the calls, too, is remarkable. Under these circumstances it is not surprising to find that one can 'phone from quite lonely spots, and that in offices and institutions it is the rule to have telephones on every floor or even in every room. There are two systems, the town system and the "national"; this accounts for a certain duplication of instruments. In hospitals there is, of course, no lack of this means of communication, and that an efficient internal system is installed goes without saying.

### The Heating Difficulty.

The heating arrangements of a large building in Sweden present a problem of greater magnitude than with us on account of the colder and longer winter. Although central heating and radiators play an important part, nevertheless the tall tiled slow-combustion stoves are to be found in most of the wards, and these are said to contribute efficiently to the warming of the ward. Special interest attaches to the means taken to obviate the obstruction to light by snow on the roof-lights of the operating theatres. In Dr. Forsner's gynæcological unit the operating theatre is, as usual, on the top floor, and is beautifully lighted. The side windows are very large and have double frames; the roof skylight is extensive and in duplicate; the space between the two lights is in winter heated by hot air, and thus snow does not get a chance of settling and blocking out the light.

### Patients' Amenities.

Provision for convalescent patients to enjoy the open air is to be found in the grounds and in the utilisation of the long promenades, which, as will be seen from the illustration, run between several of the main blocks at the height of the third storey. By means of the lifts any of the patients of any particular block can be shifted out on this "verandah," bed and all; there is plenty of accommodation for all the patients likely to benefit by outdoor rest. The patients are required to discard their own clothes and to wear suits provided by the hospital, as is also the case in Germany and in the fever hospitals of the Metropolitan Asylums Board. Patients are to be seen in the grounds of the Sabbatsberg Hospital as well as within wearing these garments.

**(The Serafina Lazaret Hospital will be described next week.)**

## Figures and Tables

**Figure f1:**
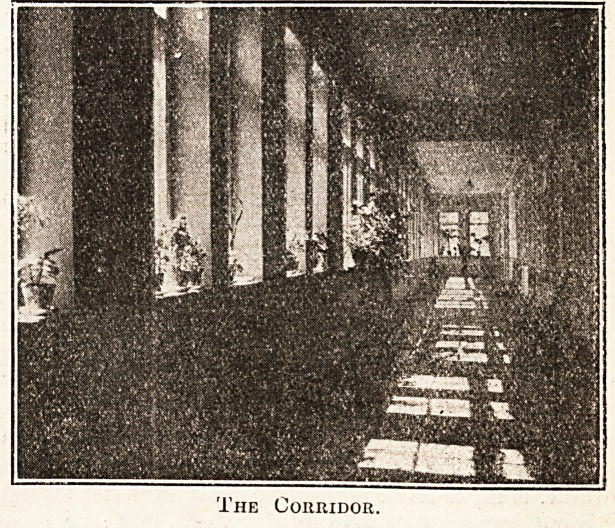


**Figure f2:**